# Supplementary material for: COVID-19 Pneumonia and Status Asthmaticus With Respiratory Failure in a Pediatric Patient: A Simulation for Emergency Medicine Providers
Source: MedEdPORTAL. 2022 Jan 21;18:11214. doi: 10.15766/mep_2374-8265.11214 (PMC8776872; doi:10.15766/mep_2374-8265.11214)
Supplement: Supplementary file 1 — Simulation Case.docxEquipment and Medication Checklist.docxLabs and Images.docxDebriefing Guide.docxSurvey.docx [file mep_2374-8265.11214-s001.zip › C. Labs and Images.docx]

VBG:

| pH | 7.34 |
| --- | --- |
| pCO_2_ | 52 mmHg |
| pO_2_ | 36 mmHg |
| HCO_3_ | 30 mEq/L |
| Base Excess | 2 mmol/L |

BMP:

| Sodium | 142 mEq/L |
| --- | --- |
| Potassium | 3.6 mEq/L |
| Chloride | 106 mEq/L |
| CO_2_ | 27 mEq/L |
| BUN | 10 mg/dL |
| Creatinine | 0.76 mg/dL |
| Glucose | 120 mg/dL |
| Lactate | 1.6 mEq/L |

CBC:

| WBC | 11.6 x 10^9^/L |
| --- | --- |
| HGB | 14.5 g/dL |
| HCT | 44.4 % |
| PLT | 214 x 10^9^/L |

Initial Chest Radiograph:


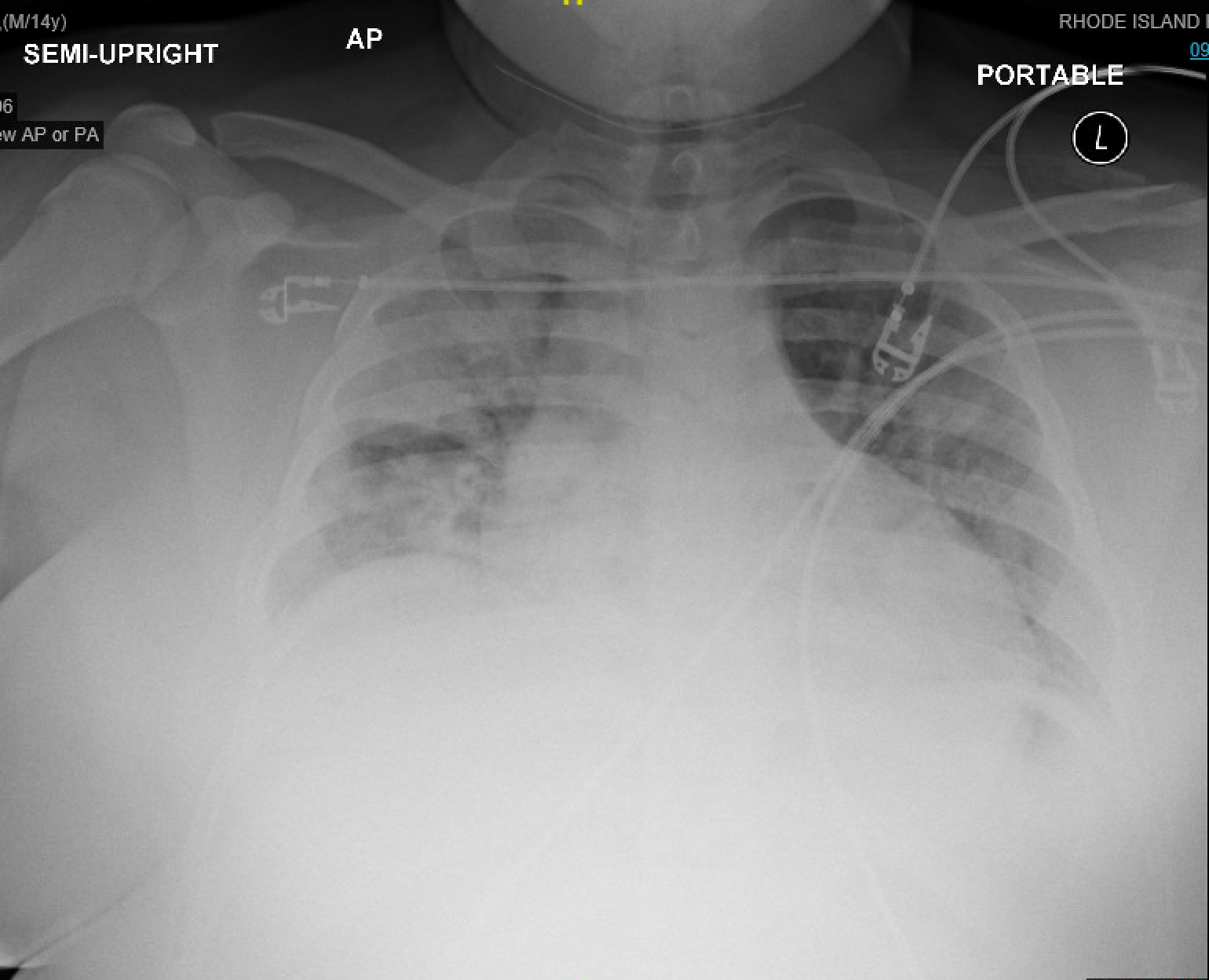


Author Owned

Post Intubation Chest Radiograph:


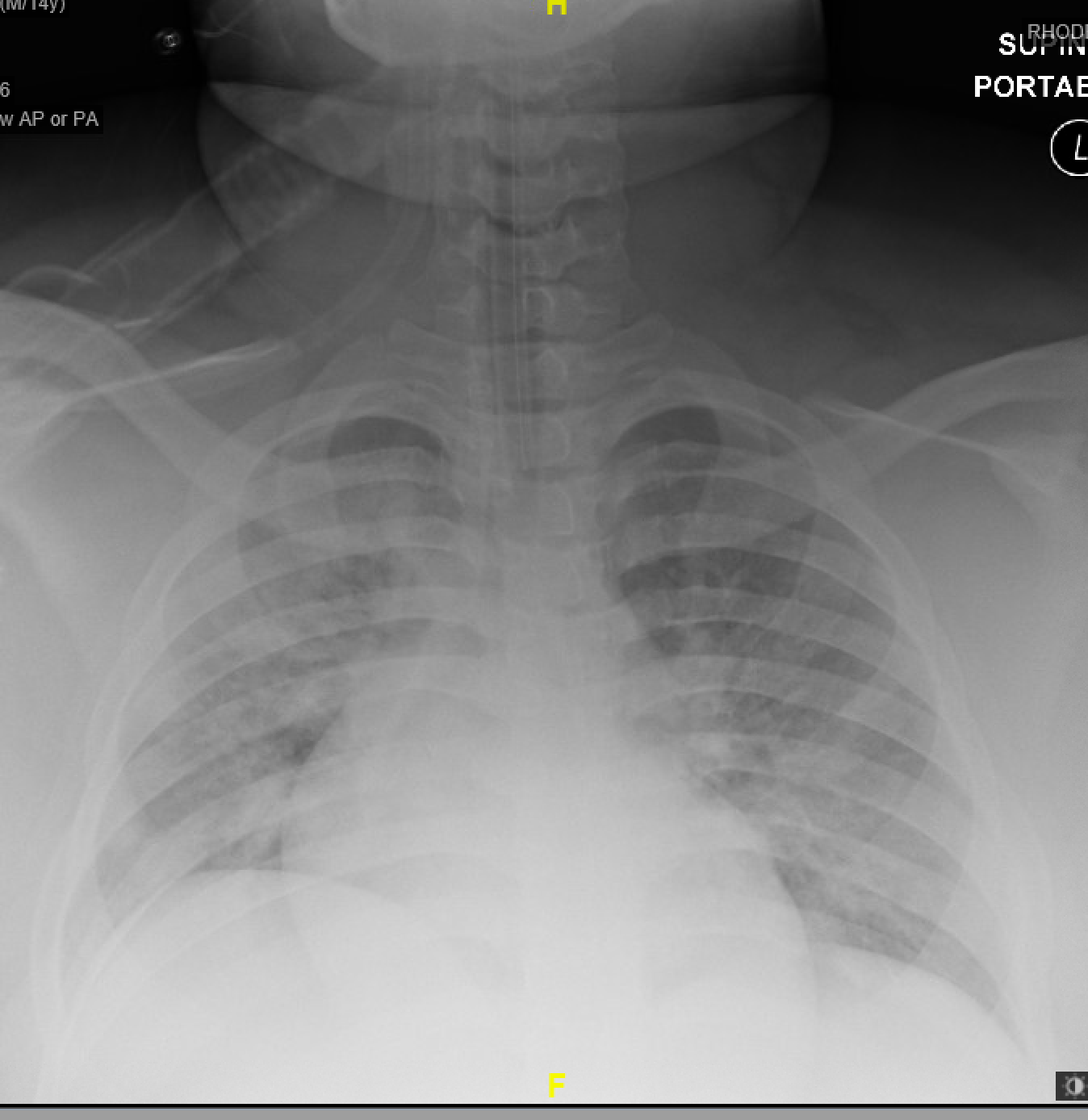


Author Owned

EKG:


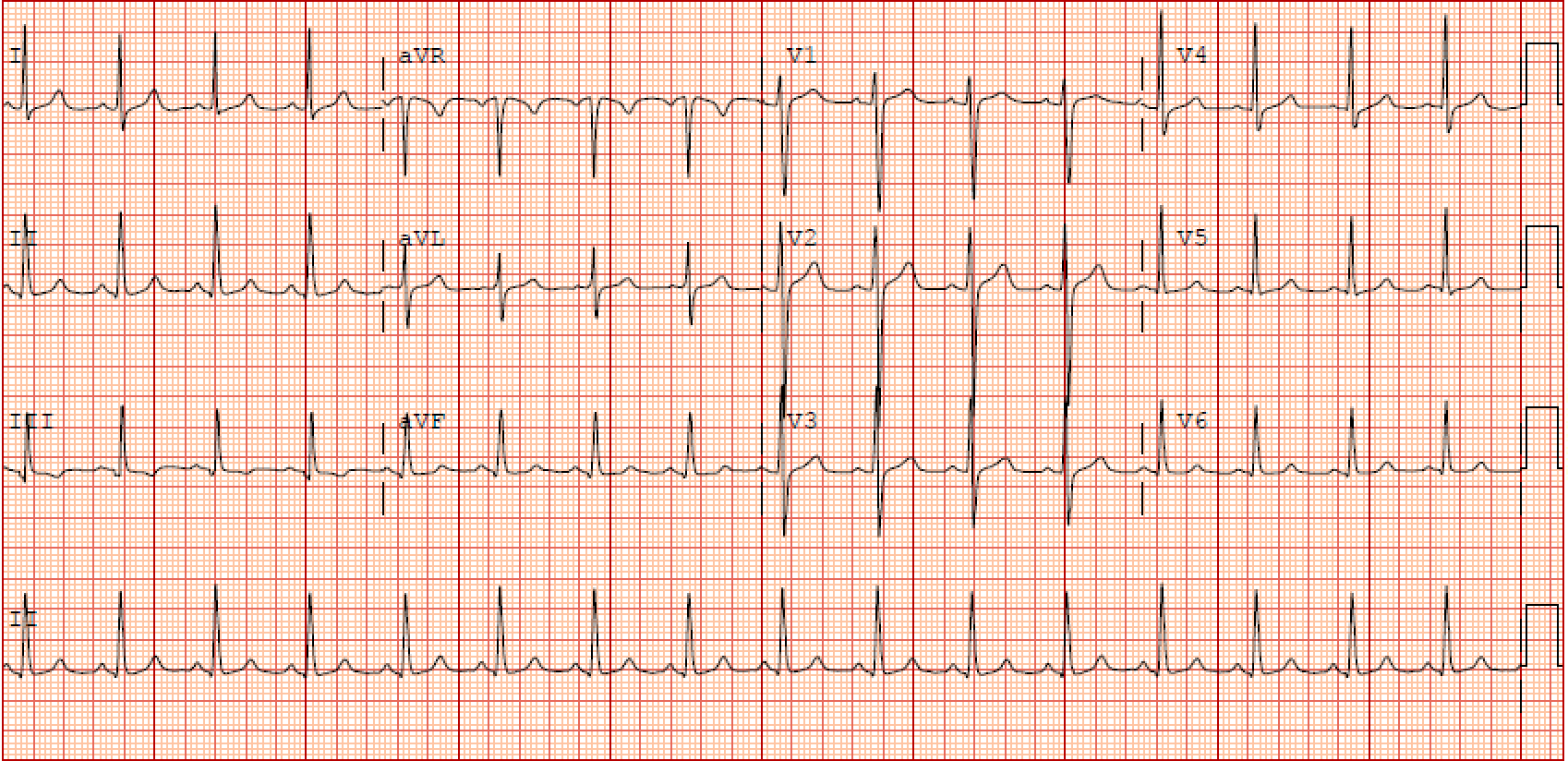


Author Owned

Interpretation: Sinus tachycardia. Intervals within normal limits.
