# Supplementary material for: COVID-19 Pneumonia and Status Asthmaticus With Respiratory Failure in a Pediatric Patient: A Simulation for Emergency Medicine Providers
Source: MedEdPORTAL. 2022 Jan 21;18:11214. doi: 10.15766/mep_2374-8265.11214 (PMC8776872; doi:10.15766/mep_2374-8265.11214)
Supplement: Supplementary file 1 — Simulation Case.docxEquipment and Medication Checklist.docxLabs and Images.docxDebriefing Guide.docxSurvey.docx [file mep_2374-8265.11214-s001.zip › D. Debriefing Guide.docx]

COVID-19 Pneumonia and Status Asthmaticus with Respiratory Failure in a Pediatric Patient - Debriefing Guide

Debriefing, an interactive, bidirectional and reflective discussion, is the most important feature of simulation-based education.^1^ There are several methods used to conduct debriefing in healthcare simulation and any would provide an adequate educational reflection for the brief scenario in our session. The key to all debriefing methods is to provide a supportive environment to ensure psychological safety, use open-ended questions to encourage active participation, and make sure to address the learning objectives. The debriefing method outlined below provides a simple yet complete structure for efficient debriefing.

Each debriefing session starts with a brief “Introduction” during which the ground rules for the debriefing session are set for all participants. This encourages a respectful and safe learning environment for optimal education and participation. The “Emotional Experience” phase of debriefing aims to encourage learners to process their emotions surrounding the case itself and/or their individual performance in the case. This is done early in the debrief in the hopes of allowing learners to move past these emotions into the remainder of the debrief. The “Teamwork & Communication” phase focuses on examining team dynamics and how that affected patient care. The “Medical Management” phase allows the team to review the facts of the case and solidify many of the cases learning objectives. Lastly, the “Wrap Up” phase encourages learners to highlight their take home points and gives facilitators a final opportunity to ensure that learning objectives have been covered.

| Debriefing Guide:  (Please refer to the Learning Objectives and Critical Actions to help guide discussion) | |  |
| --- | --- | --- |
| **Debriefing Phase** | Suggested questions/phrases | Case-Specific Discussion Points & Reference Materials |
| **Introduction** | We are now going to take the next 15 to 25 minutes to debrief. This part of our session is more important than the actual case. As a reminder, our ground rules are as follows:   - 1. This is meant to be a safe learning environment   2. Simulation is a formative, interdisciplinary learning environment - no one is being evaluated (except the simulation instructors because we constantly evaluate ourselves on how to provide better educational sessions).   3. We all agree to the basic assumption that everyone here is intelligent, well trained, and cares about doing their best.   4. Thus, we all agree to the highest standard of professional conduct and courtesy to our colleagues in this debrief and in any further discussions about this mock code. |  |
| **Emotional Experience** | - That was a challenging/stressful/difficult case. Let’s talk about what just happened. - What part of this scenario was particularly challenging? Why? | - Validate learner’s reactions and emotions - Invite thoughts from several learners |
| **Teamwork & Communication** | - Who were the members of the care team and how were roles established or assigned? | - Team leader – Identifies leader role, clear direction, team coordination, invites feedback, stands at foot of bed - Survey/Airway MD/DO - Primary and secondary survey, communicates back to team leader, frequent reassessment, manages airway, prepares for advanced airway if necessary, stands at head of bed - Bedside/Medication Nurse (Confederate) – Obtains access, draws up medications as ordered, administers medications, call backs to team leader |
|  | - What elements of team communication went well? - What could be improved upon? - Did everyone know what the team leader was thinking? - Did everyone on the team feel empowered to speak up to offer suggestions or additions to patient care? | Find examples of/suggest examples of the following:   - Closed loop communication – receivers of information confirm information to the sender   - *Ex. Team leader – “Jennifer, please place an IV in the patient”; Jennifer – “Placing an IV in the patient”* - Directed call outs – using team members’ names or specific roles to ensure orders/assignments are not missed   - *Ex. “John, please obtain a manual blood pressure”; “Team leader, I am having difficulty obtaining a manual blood pressure.”* - Shared mental model with periodic case re-summarization to assure that all team members know the working diagnosis, management priorities and next steps for patient care.   - *Ex. “Given the patient’s respiratory distress and altered mental status, I am concerned about [X]. Our priorities should be placing the patient on oxygen and obtaining IV access.”* - Team members welcomed to participate in management decisions   - *Ex. “Dr. Smith, I am concerned that we should limit personnel in the room if possible as this patient may have COVID-19.” “I am concerned that this patent may have a difficult airway. My plan is to call for backup resources including anesthesia and PICU. Does everyone agree?”* |
| **Medical Management** | - What happened when you first entered the room? - What led to the recognition of respiratory failure? | - C, A, Bs should be quickly assessed - Recognize that this patient could potentially have COVID-19   - Ensure that all team members and learners in the room have appropriate PPE on (N95, goggles, face shield, gown, gloves^2^)   - Limit number of personnel in room to the most experienced/essential providers to minimize infection exposure - Venous blood gas with mild respiratory acidosis (would have expected much lower pCO_2_ given severe work of breathing) - Hypoxemia despite high concentrations of oxygen or non-invasive positive pressure ventilation (NIPPV) - Severe increased work of breathing (e.g. inability to speak) - Altered mental status - Hypercarbia alone is not an indication for intubation, but intubation is needed if PaCO_2_ continues to rise despite medical therapy +/- NIPPV, or if hypercarbia is causing significant respiratory acidosis or altered mental status^3,4^ |
|  | - What was your initial differential diagnosis? | - Pneumonia (acute COVID-19 infection, bacterial, other viral) - Multisystem inflammatory syndrome in children (MIS-C) - Status asthmaticus - Pulmonary embolism - Congestive heart failure - Pneumothorax - Acute respiratory distress syndrome - Metabolic acidosis with respiratory compensation |
|  | - What are ways to distinguish between these diagnoses? | - Symptoms of acute COVID-19 infection include cough, fever, myalgia, headache and dyspnea.^5^ Pneumonia is the most frequent serious manifestation of acute COVID-19 infection.^6^ - The diagnosis of MIS-C requires positive confirmed current or recent SARS-CoV-2 infection, or exposure to a suspected or confirmed COVID-19 case within 4 weeks prior to symptom onset. Symptoms typically include abdominal pain, vomiting, diarrhea, rash, hypotension and shock in addition to fever. Workup may also reveal cardiac dysfunction, myocarditis or acute kidney injury.^7^ - Clinical improvement with standard asthma therapies (as discussed below) suggests status asthmaticus - Consider using POCUS echocardiogram to evaluate for CHF (decreased squeeze, plethoric IVC) - Consider using imaging modalities such as chest radiograph or POCUS lung to evaluate for pneumonia (loss of lung sliding, B-lines, dynamic air bronchograms, pulmonary “hepatization” or liver-like echogenicity on POCUS lung) vs. CHF (at least 3 B-lines in a rib space in at least 2 lung zones bilaterally on POCUS lung) |
|  | - Why/when did you suspect COVID-19 pneumonia? | - Vital sign abnormalities - Respiratory exam with increased work of breathing and hypoxemia |

|  | - After [____], what happened next? - After [____], how did the team respond? - I noticed you chose to [____], tell us about your thought process at that point. | - Consider escalation of respiratory support - Consider IV fluid and electrolyte repletion as needed - Draw blood cultures and give antibiotics empirically for bacterial pneumonia in a sick patient - Obtain chest radiograph - Monitor and reassess vital signs frequently |
| --- | --- | --- |
|  | - What resources did you mobilize (or could you have called upon) to manage this potential COVID-19 positive patient in the Emergency Department? | - Institution-specific: Anesthesia, PICU, airway team, intubation and/or COVID-19 checklist/protocols etc. - How specifically do you obtain these consultations and/or resources at your institution? How do these resources change overnight or during different times of the day? |
|  | - What is the management for a patient with status asthmaticus? | - Albuterol - Ipratropium - Magnesium with IV fluids - IM Epinephrine - Terbutaline - BiPAP - Intubation as last resort given challenges of worsened airway obstruction, impaired exhalation with worsening of hypercarbia and risk of barotrauma. - Intubation induction sedation agents to consider specifically in patients with asthma include ketamine or propofol, as these medications have bronchodilation effects.^8^ Ketamine is generally preferred over propofol because propofol can cause hypotension, which is non-ideal in patients with asthma who are already predisposed to hypotension after intubation. Paralytics such as succinylcholine or rocuronium can be used in patients with asthma. |
| **Wrap Up** | - What unanswered questions do you have about this case? - What is your take home point? (go around the room and have every learner state one) |  |

References:

1. Sawyer T, Eppich W, Brett-Fleegler M, Grant V, Cheng A. More than one way to debrief: a critical review of healthcare simulation debriefing methods. *Simul Healthc.* 2016;11(3):209-217.

2. Organization WH. *Rational use of personal protective equipment (PPE) for coronavirus disease (COVID-19): interim guidance, 19 March 2020.* World Health Organization; 2020.

3. Asthma: a follow up statement from an international paediatric asthma consensus group. *Arch Dis Child.* 1992;67(2):240-248.

4. Qureshi F. Management of children with acute asthma in the emergency department. *Pediatr Emerg Care.* 1999;15(3):206-214.

5. Stokes EK, Zambrano LD, Anderson KN, et al. Coronavirus Disease 2019 Case Surveillance - United States, January 22-May 30, 2020. *MMWR Morb Mortal Wkly Rep.* 2020;69(24):759-765.

6. Guan WJ, Ni ZY, Hu Y, et al. Clinical characteristics of coronavirus disease 2019 in China. *N Engl J Med.* 2020;382(18):1708-1720.

7. Centers for Disease Control and Prevention. Information for healthcare providers about multisystem inflammatory syndrome in children (MIS-C). <https://www.cdc.gov/mis/hcp/index.html>. Published 2021. Accessed 9 September, 2021.

8. Brenner B, Corbridge T, Kazzi A. Intubation and mechanical ventilation of the asthmatic patient in respiratory failure. *Proc Am Thorac Soc.* 2009;6(4):371-379.
