# Supplementary material for: COVID-19 Pneumonia and Status Asthmaticus With Respiratory Failure in a Pediatric Patient: A Simulation for Emergency Medicine Providers
Source: MedEdPORTAL. 2022 Jan 21;18:11214. doi: 10.15766/mep_2374-8265.11214 (PMC8776872; doi:10.15766/mep_2374-8265.11214)
Supplement: Supplementary file 1 — Simulation Case.docxEquipment and Medication Checklist.docxLabs and Images.docxDebriefing Guide.docxSurvey.docx [file mep_2374-8265.11214-s001.zip › B. Equipment and Medication Checklist.docx]

**COVID-19 Pneumonia and Status Asthmaticus with Respiratory Failure in a Pediatric Patient - Simulation Scenario Equipment and Medication Checklist**

This checklist is to be used to ensure a high-fidelity stimulation experience for learners. While the following list contains items commonly found in most emergency departments to care for critically ill pediatric patients, these resources, medications and equipment may need to be modified to reflect what is available to learners in practice in their clinical environments.

**RESOURCES**

☐Pediatric Advanced Life Support (PALS) reference cards

☐Broselow Tape

☐Institution Specific Resources (may or may not be pertinent and/or available at specific institution)

☐Pediatric Medication Book

☐Pediatric Intubation Checklist

☐PPE Policy (may or may not be available at specific institution)

☐Management of COVID-19 positive patient protocol/policy (may or may not be available at specific institution)

**PERSONAL PROTECTIVE EQUIPMENT**

☐Gloves (all sizes available for participants)

☐Gown

☐Eye protection such as goggles

☐Face shield

☐N95 mask

☐Controlled Air Purifying Respiratory (CAPR) or Powered Air Purifying Respirator (PAPR) (if available at institution)

**MANNIKIN**

☐High-fidelity manikin that allows for assessment of chest rise with bag-valve-mask and intubation

☐Gown

☐Padding under gown

☐Sheet

**PEDIATRIC SIMULATION EQUIPMENT**

☐Monitor (HR, RR, oxygen saturation, blood pressure, temperature and EtCO_2_ monitor)

☐Heart rate monitor leads, oxygen saturation probe, blood pressure cuff (multiple sizes), EtCO_2_ cannula

☐Oxygen hook-up on wall or cylinder

☐Suction cannister, tubing, and Yankauer suction tip

☐Stethoscopes (one for each provider if available)

☐Towels

☐Oxygen delivery devices: nonrebreathers, nasal cannulas, BVMs (multiple sizes)

☐Intubation Tray:

☐Miller blade sizes 2, 3, 4 – minimum requirement: sized to the manikin

☐Macintosh blade sizes 2, 3, 4 – minimum requirement: sized to the manikin

☐Cuffed endotracheal tubes (ETTs) sizes 6.0, 6.5, 7.0, 7.5, 8.0 – minimum requirement: sized to the manikin

☐Adult and pediatric stylets – minimum requirement: sized to the manikin

☐LMA sizes 2.5, 3, 4, 5 – minimum requirement: sized to the manikin

☐Adult and pediatric size end-tidal CO_2_ colorimeters – minimum requirement: sized to the manikin

☐Syringe

☐Tape

☐Tracheal tube introducer such as a bougie (pediatric and adult sizes) – minimum requirement: sized to the manikin

☐Endotracheal tube HEPA filter (if available at institution)

☐Nasopharyngeal and oropharyngeal airways (multiple sizes) – minimum requirement: sized to the manikin

☐Video laryngoscopy device, stylet and handles (multiple sizes) – minimum requirement: sized to the manikin

☐Nasogastric tubes (multiple sizes) – minimum requirement: sized to the manikin

☐Intravenous (IV) Supplies:

☐IV/Angiocath, various sizes

☐Tourniquet

☐Syringes

☐IV pole and pump

☐IV tubing and filters

☐Pressure bags

☐Specimen tubes

☐Gauze and Tape

☐Sharps Container

☐Respiratory Medication Supplies

☐Metered Dose Inhaler

☐Spacer

☐Nebulizer

☐Code cart with:

☐Defibrillator

☐Defibrillator pads (pediatric and adult size) – minimum requirement: sized to the manikin

☐Backboard

☐Step Stool

**MEDICATIONS**

☐Acetaminophen

☐Albuterol

☐Albuterol-ipratropium

☐Ceftriaxone

☐Dexamethasone

☐Epinephrine 1:1,000

☐Epinephrine 1:10,000

☐Etomidate

☐Fentanyl

☐Ibuprofen

☐Ipratropium

☐Heliox

☐Hydrocortisone

☐Ketamine

☐Lidocaine

☐Lorazepam

☐Magnesium

☐Midazolam

☐Normal Saline/Lactated Ringers

☐Rocuronium

☐Solumedrol

☐Succinylcholine

☐Terbutaline

☐Vancomycin
