# Supplementary material for: COVID-19 Pneumonia and Status Asthmaticus With Respiratory Failure in a Pediatric Patient: A Simulation for Emergency Medicine Providers
Source: MedEdPORTAL. 2022 Jan 21;18:11214. doi: 10.15766/mep_2374-8265.11214 (PMC8776872; doi:10.15766/mep_2374-8265.11214)
Supplement: Supplementary file 1 — Simulation Case.docxEquipment and Medication Checklist.docxLabs and Images.docxDebriefing Guide.docxSurvey.docx [file mep_2374-8265.11214-s001.zip › A. Simulation Case.docx]

| **Appendix A: COVID-19 Pneumonia and Status Asthmaticus with Respiratory Failure in a Pediatric Patient Simulation Case**  **SIMULATION CASE TITLE**: 13-year-old male with status asthmaticus, COVID-19 pneumonia and respiratory failure  **AUTHORS:** Hoi See Tsao, MD, MPH & Robyn Wing, MD, MPH  **LEARNER AUDIENCE:** Emergency Medicine Residents, Pediatric Emergency Medicine Fellows | |
| --- | --- |
| **PATIENT NAME**: Edward Brown  **PATIENT AGE:** 13 years old  **CHIEF COMPLAINT:** Difficulty breathing  **PHYSICAL SETTING:** (Pediatric) Emergency Department | |
|  | |
| **Brief narrative description of case** | 13-year-old male with obesity and asthma presents with shortness of breath in the setting of a COVID-19 positive contact. The patient quickly decompensates in the emergency department (ED) and requires endotracheal intubation. |
| **Learning Objectives** | By the end of this activity, learners will be able to:   1. Establish a differential diagnosis for respiratory distress in a potentially COVID-19-positive pediatric patient. 2. Demonstrate the management of status asthmaticus in a pediatric patient. 3. Identify acute respiratory failure. 4. Anticipate and plan for a difficult airway in a potentially COVID-19-positive patient. 5. Demonstrate the appropriate use of personal protective equipment and resources for aerosolizing procedures with a potentially COVID-19-positive patient. |
| **Critical Actions** | 1. Identify patient as a Person Under Investigation (PUI) and don appropriate PPE 2. Verbalize infection exposure concerns to prompt healthcare team members to also don appropriate PPE 3. Assess C, A, Bs including the following: 4. Place patient on full cardiac monitor 5. Recognize vital sign abnormalities of tachypnea, hypoxia and tachycardia 6. Administer oxygen 7. Request IV access 8. Obtain labs including venous blood gas, CBC, blood culture 9. Trial bronchodilator therapy and consider IM Epinephrine 10. Limit personnel in the room when treating with aerosolizing treatments or procedures 11. If necessary, move patient to a negative pressure room or room with a HEPA filter 12. Identify potential need for advanced respiratory support and potential difficult airway 13. Effectively communicate concerns for respiratory failure with healthcare team 14. Prepare intubation equipment 15. Call for respiratory therapy and consider calling PICU or anesthesia for support 16. Obtain chest radiograph 17. Intubate 18. Consider broad-spectrum antibiotics such as ceftriaxone 19. Stabilize and transfer to PICU |
| **Learner Preparation or Prework** | Assure learners of psychological safety during and after their participation. This brief simulation is intended to be formative and is solely for learning purposes.  The facilitators orient learners to the manikin, encourage them to treat him like a real live patient, and encourage learners to ask questions if they are unsure of something due to the simulation setting. Learners are informed that they will be working as a team to care for this patient.  The facilitators describe the setting for this scenario as follows:  Edward, a 13-yr-old male with obesity and asthma, presents with shortness of breath in the setting of a COVID-19 positive contact. |

| Initial Presentation | | | | | |  |
| --- | --- | --- | --- | --- | --- | --- |
| **Initial vital signs** | | HR:118  RR: 74  BP: 161/88  SaO_2_ 97% on 4L NC  Temp: 102^o^F  Wt: 160 kg | | | | |
| **Overall Setting and Appearance** | | The patient is obese and appears to be in severe respiratory distress with tripod-positioning, nasal flaring and suprasternal retractions. | | | | |
| **Confederates (e.g., standardized participants) and their roles in the room at case start** | | If there is only one confederate running the simulation, the following roles can be combined and performed by one confederate facilitator.  **EMS/Faculty Instructor:** EMS provider is appropriate and helpful but appears stressed. EMS was called to the house due to respiratory distress. The patient’s mom told EMS that the patient has asthma. Mom gave him many puffs of his albuterol inhaler with a spacer, but the medication did not help. After presenting the history by EMS, the confederate then takes the role of faculty instructor. As faculty instructor, this confederate would be present, either in the control room for simulation equipment, or in person (if also playing the role of **Nurse**). He/she verbalizes physical examination findings (such as rales, irregular heart rhythm, etc.). upon observing the learners perform that aspect of the exam on the manikin. The faculty instructor observes the performance of the learner(s), provides feedback and instruction to the nurse to facilitate case progression, and facilitates the debriefing session.  **Nurse:** The nurse is available to answer questions regarding the patient’s history and events leading up to the current emergency department presentation. The nurse is helpful but very uncomfortable with this sick patient. The nurse asks questions about what therapies he/she is “allowed” to administer due to potential COVID-19 infection. “I don’t think we are allowed to give nebulizer treatments anymore, are we?” If the team leader gives a good explanation, the nurse accepts the explanation and proceeds with orders. | | | | |
| **HPI** | | 13-year-old male with obesity and asthma presenting via EMS from home for evaluation of difficulty breathing. The patient was seen by his primary care pediatrician two days ago for congestion, cough and fever. At that time, he was given a prescription for prednisone. A COVID-19 test was sent, but the results are not back yet. He has been using albuterol at home “a lot” with only transient effect. Patient reports that he woke up in the middle of the night with significantly increased shortness of breath and his family called EMS.  EMS reports oxygen saturations of 88-90% on room air on their arrival. They placed the patient on 4 L of O_2_ via nasal cannula with improvement of his oxygen saturation to 95%. They report significantly increased work of breathing and tachypnea during transport.  The following details will be revealed only when asked by the learner:   - Patient has had 5 days of nasal congestion, dry cough and fever with a T-max of 103. - Patient has had decreased oral intake but has normal urine output. - Patient denies any nausea, vomiting or diarrhea. - Patient reports his aunt, who lives in the home, has COVID-19 currently. | | | | |
| **Past Medical/Surgical History** | | **Medications** | **Allergies** | **Family History** | | |
| Asthma – no hospitalizations or intubations  Obesity | | Albuterol PRN | None Known | Aunt with COVID-19 | | |
| **Physical Examination** | | | | | |  |
| **General** | Obese, in severe respiratory distress, unable to complete full sentences, in tripod position | | | |  |  |
| **HEENT** | Conjunctiva normal, PERRLA, nares congested, mucous membranes moist, oropharynx clear | | | |  |  |
| **Neck** | Full range of motion, no lymphadenopathy | | | |  |  |
| **Lungs** | Tachypnea, in obvious respiratory distress, + wheeze throughout, crackles in RLL | | | |  |  |
| **Cardiovascular** | Tachycardic, regular, no m/r/g | | | |  |  |
| **Abdomen** | Soft, nontender, nondistended | | | |  |  |
| **Neurological** | Alert and oriented, grossly intact, able to follow commands | | | |  |  |
| **Skin** | Warm and well perfused, no rashes | | | |  |  |
| **Musculoskeletal** | No swelling, tenderness or signs of injury | | | |  |  |
| **Psychiatric** | Agitated, anxious, repetitive | | | |  |  |

| Instructor Notes - Changes and CASE Branch Points | | |
| --- | --- | --- |
| Intervention / Time point | Change in Case | Additional Information |
| Patient placed on cardiopulmonary monitor, vital signs obtained, IV access obtained | If yes 🡪 | Vital signs displayed on monitor:  HR: 118/min  BP: 161/88  RR: 74/min  O_2_ sat: 95 % on 4L NC  T: 102^o^F |
|  | If no 🡪 | Nurse can ask, “Would you like me to put this patient on a monitor?” and/or “Would you like me to obtain IV access?” |
|  | If still no 🡪 | Nurse says, “I’m going to put him on a monitor” and/or “I’m going to obtain IV access.” |
| Patient is placed on a nonrebreather mask | If yes 🡪 | Oxygen level increases to 97%. |
|  | If no 🡪 | Oxygen level stays at 95% on 4L NC. |
| Obtain pertinent history | If yes 🡪 | Continue the case. Faculty instructor will verbalize findings. |
|  | If no 🡪 | Patient/parent offer pertinent history. |
| Checks that team is in appropriate PPE | If yes 🡪 | Case continues. |
|  | If no 🡪 | Nurse says, “I am just going to wear my surgical mask. Is that okay?” |
| Minimizes personnel in room (if there are more than 2 learners actively participating in the case) | If yes 🡪 | Case continues. |
|  | If no 🡪 | Nurse prompts “Do we need this many people in here?” |
| Bloodwork is requested | If yes 🡪 | Point of care venous blood gas available a few minutes after request.  BMP, CBC and lactate available several minutes after request.  Any other requested labs are sent to lab and pending.  See Appendix C. |
|  | If no 🡪 | Nurse prompts, “Would you like me to obtain any bloodwork?” |
| Chest radiograph (CXR) is requested | If yes 🡪 | CXR demonstrates multifocal patchy airspace opacities bilaterally.  See Appendix C. |
|  | If no 🡪 | Nurse prompts, “Would you like me to call for CXR?” |
| Electrocardiogram (EKG) is requested | If yes 🡪 | EKG demonstrates sinus tachycardia with normal intervals.  See Appendix C. |
|  | If no 🡪 | Nurse prompts, “Would you like me to perform an EKG?” |
| Learners verbalize their interpretation of labs, CXR and EKG | If yes 🡪 | Case continues. |
|  | If no 🡪 | Nurse asks what learners think of these tests. |
| Normal saline (NS) IV bolus is requested and administered | If yes or if no 🡪 | Patient has no change in symptoms. Vitals remain unchanged. |
| Antipyretics (ibuprofen or acetaminophen) is requested and administered | If yes 🡪 | Case continues. After 10 min, nurse asks, “Do you want me to take a repeat temperature?”  After 10 minutes, temperature is 98.3^o^F. |
|  | If no 🡪 | Case continues. |
| Antibiotics requested and administered | If yes 🡪 | Case continues. |
|  | If no 🡪 | Nurse asks if the learners want any additional medications administered. |
| Bronchodilator therapy (albuterol-containing inhalers or nebulizers) and/or IM epinephrine requested and administered within first 3 minutes of case | If yes 🡪 | If nebulizers requested, nurse asks, “I don’t think we are allowed to give nebulizer treatments, are we?” If the learner gives a reasonable explanation, nurse administers medication requested and case continues.  Vital signs displayed on monitor.  HR: 140/min  BP: 150/90  RR: 60/min  O_2_ sat: 95% on 4L (or any O_2_) |

|  | If no 🡪 | Case progresses to respiratory failure with the following vitals displayed on monitor.  HR: 150/min  BP: 150/90  RR: trends down to 20/min  O_2_ sat: trends down to 80% on NRB, or 60% on any other type of O_2_ |
| --- | --- | --- |
| Magnesium and/or terbutaline requested and administered within first 6 minutes of case | If yes or if no 🡪 | Case progresses to respiratory failure with the following vitals displayed on monitor.  HR: 150/min  BP: 150/90  RR: trends down to 20/min  O_2_ sat: trends down to 80% on NRB, or 60% on any other type of O_2_ |
| Learners verbalize anticipation of a difficult airway | If yes 🡪 | Case continues. |
|  | If no 🡪 | Nurse prompts, “Will this be an easy airway?” and/or “Do you think that you will need any help for intubation?” |
| Learners verbalize preparation of difficult airway supplies, such as video/direct laryngoscopy, LMA and/or tracheal tube introducer such as a bougie | If yes 🡪 | Case continues. |
|  | If no 🡪 | Nurse prompts, “Is there any equipment we will need for intubation? |
| Learners request PICU or Anesthesia for help during intubation | If yes 🡪 | Nurse responds, “They have been paged but are managing a code in the hospital. They will be here as soon as possible.” |
|  | If no 🡪 | Case continues. |
| Learners prepare for advanced respiratory support (CPAP, BiPAP or intubation) within first 8 minutes of case | If yes 🡪 | Case continues. |
|  | If no intubation 🡪 | Nurse prompts, “I am worried about this patient’s respiratory status. Is there anything we should do?”  Case progresses to asystolic arrest if intubation is not performed. ROSC obtained after 1 dose of epinephrine.  Vital signs on monitor displayed during asystolic arrest:  HR: 0  BP: Unable to obtain  RR: 0  O_2_ sat: Slow trend down to 0% |
| Post-intubation chest radiograph (CXR) is requested | If yes 🡪 | CXR demonstrates “The endotracheal tube tip is in the proximal thoracic trachea near the thoracic inlet. Improved aeration of the right upper lobe. Low lung volumes with residual multifocal pulmonary opacities.”  Nurse prompts, “What does the CXR show?”  See Appendix C. |
|  | If no 🡪 | Nurse prompts, “Do you want me to call for CXR?” |
| The patient is admitted and/or transferred to the intensive care unit | If yes 🡪 | The case concludes. |
|  | If no 🡪 | Nurse prompts, “Did you already request a bed for this patient?” |

**Ideal Scenario Flow**

At the start of the simulation, participants are informed by emergency medical services (EMS) that they brought in a patient with a history of asthma in respiratory distress. He was not improving despite the use of the albuterol inhaler his mother gave him at home. EMS noted an oxygen saturation of 88% on room air and placed the patient on 4L of oxygen via nasal cannula en route. The participants don the appropriate PPE prior to entering the room. The participants enter to see a patient with obesity in respiratory distress. During history-taking, participants discover that the patient lives with an aunt who has COVID-19. Participants ensure that the nurse has appropriate PPE on and instruct the nurse to place the patient on the full cardiac monitor and obtain IV access and labs. Participants recognize the patient’s abnormal vital signs (tachycardia, tachypnea and fever). They perform a physical examination and find the patient in severe respiratory distress, unable to complete full sentences, tripod-positioning, and with diffuse wheeze and crackles in the right lower lobe.

Bloodwork (venous blood gas, BMP, CBC, lactate) and chest radiograph are ordered that show a mild respiratory acidosis (that is inappropriate given the patient’s severe work of breathing) and multifocal patchy airspace opacities bilaterally. Participants ask for status asthmaticus and pneumonia therapies including albuterol-containing inhalers or nebulizers, steroids, intramuscular epinephrine, magnesium, terbutaline, antibiotics and non-invasive ventilatory support devices such as BiPAP. The nurse asks if therapies such as nebulizers are allowed during COVID-19 and participants reassure the nurse of the necessity of these medications given the patient’s deteriorating clinical status.

The patient progresses to respiratory failure. Participants recognize that the patient will likely have a difficult airway due to the patient’s obesity, status asthmaticus, and potential COVID-19 infection. In preparing for a likely difficult intubation, participants recognize that this is an aerosol-generating procedure and decrease the number of personnel in the room (if there are more than 2 learners actively participating), have an experienced provider with appropriate PPE on intubate with an endotracheal tube filter (depending on institutional resources), and consider calling the pediatric intensive care unit (PICU) or anesthesia early. The patient is then admitted to the PICU.

**Anticipated Management Mistakes**

Specific prompts and responses for anticipated management mistakes are outlined in the “Instructor notes- changes and branch points” section above. To summarize, they include:

- Failure to obtain pertinent COVID-19 exposure history. Some learners did not obtain the exposure history of the patient living with his aunt who has COVID-19. This history element that learners failed to obtain during the simulation should be discussed in the debriefing session. In addition, given the patient’s symptomatology and even without the COVID-19 exposure history, learners should recognize that this is patient who potentially has COVID-19.
- Failure to recognize acute respiratory distress and hypoxia. If learners fail to recognize the patient’s respiratory distress and hypoxia, the confederate nurse should verbally bring attention to the patient’s distress and hypoxia. If learners fail to recognize these signs in a timely manner, this should be discussed in the debriefing session.
- Failure to treat status asthmaticus. If learners fail to recognize the pertinent exam findings of diffuse wheeze and respiratory distress in a patient with a history of asthma suggestive of status asthmaticus, the confederate nurse can prompt by asking the learners what diagnoses they are considering and/or if they would like additional medications given. Any physical examination elements that learners fail to identify during the simulation should be discussed in the debriefing session.
- Failure to workup and treat pneumonia. If the learners fail to recognize the pertinent exam findings of fever, right lower lobe crackles and respiratory distress suggestive of pneumonia, the confederate nurse can prompt by asking the learners what diagnoses they are considering and/or if they would like additional lab work drawn or medications given. Any physical exam elements that the learners fail to identify during the simulation should be discussed in the debriefing session.
- Failure to anticipate and plan for a difficult airway. If the learners fail to recognize that the patient with obesity, status asthmaticus and likely COVID-19 pneumonia potentially has a difficult airway, the confederate nurse can prompt by asking learners “Will this be an easy airway?” and/or “Do you think that you will need any help for intubation?” Padding under the patient’s gown was also placed on the manikin to simulate obesity. Additional institutional-specific (such as an “airway team” if available) and difficult airway resources should be discussed in the debriefing session.
- Failure to demonstrate appropriate use of PPE during aerosolizing procedure in a potential COVID-19 positive patient. Learners should recognize that given the patient’s symptomatology (and the positive COVID-19 exposure history if elicited), the patient may have COVID-19 and should don appropriate PPE. If learners fail to don appropriate PPE, this should be discussed in the debriefing session.
- Failure to manage resources to minimize COVID-19 exposure among healthcare providers and other patients/families. In a patient with potential COVID-19, learners should minimize the number of personnel in the room to the most experienced providers including having the most experienced person intubate the patient and ensure that everyone in the room is wearing appropriate PPE. The confederate nurse can prompt by saying, “I am just going to wear my surgical mask. Is that okay?” and/or “Do we need this many people in here?” If learners fail in these elements, this should be discussed in the debriefing session, including emphasis on the learners’ roles as team leaders in keeping their healthcare team safe.
