# Supplementary material for: COVID-19 Pneumonia and Status Asthmaticus With Respiratory Failure in a Pediatric Patient: A Simulation for Emergency Medicine Providers
Source: MedEdPORTAL. 2022 Jan 21;18:11214. doi: 10.15766/mep_2374-8265.11214 (PMC8776872; doi:10.15766/mep_2374-8265.11214)
Supplement: Supplementary file 1 — Simulation Case.docxEquipment and Medication Checklist.docxLabs and Images.docxDebriefing Guide.docxSurvey.docx [file mep_2374-8265.11214-s001.zip › E. Survey.docx]

**COVID-19 Pneumonia and Status Asthmaticus with Respiratory Failure in a Pediatric Patient:**

**Case Survey**

1. What is your current level of training?

☐ PGY 1 ☐ PGY 2 ☐ PGY 3 ☐ PGY 4

☐ PGY 5 ☐ PGY 6 ☐ PGY 7 ☐ Attending Physician

☐ Other _______________________

1. If you are a resident, what residency program are you in?

☐ Emergency Medicine ☐ Pediatrics ☐ Other _______________________

1. Are you participating in this session in-person or virtually?

☐ In-person ☐ Virtually ☐ Other _______________________

**Please rate your agreement with the following statements:**

|  |  | Strongly Disagree | Disagree | Neither Agree nor Disagree | Agree | Strongly Agree |
| --- | --- | --- | --- | --- | --- | --- |
| 3. | This simulation case provided is relative to my work. | □ | □ | □ | □ | □ |
| 4. | This simulation case was realistic. | □ | □ | □ | □ | □ |
| 5. | This simulation case was effective in teaching the evaluation and management of respiratory failure in the time of COVID-19. | □ | □ | □ | □ | □ |
| 6. | The debrief created a safe environment. | □ | □ | □ | □ | □ |
| 7. | The debrief promoted reflection and team discussion. | □ | □ | □ | □ | □ |

**After participating in this session, how confident are you in your ability to:**

|  |  | Very Not confident | Not confident | Neutral | Confident | Very Confident |
| --- | --- | --- | --- | --- | --- | --- |
| 8. | Manage status asthmaticus. | □ | □ | □ | □ | □ |
| 9. | Evaluate the causes of respiratory distress in a potentially COVID-19-positive patient. | □ | □ | □ | □ | □ |
| 10 | Evaluate for causes of respiratory distress in a patient with asthma in the time of COVID-19. | □ | □ | □ | □ | □ |
| 11 | Identify acute respiratory failure. | □ | □ | □ | □ | □ |
| 12 | Anticipate and plan for a difficult airway. | □ | □ | □ | □ | □ |
| 13 | Demonstrate appropriate use of personal protective equipment for an aerosolizing procedure in a potentially COVID-19-positive patient. | □ | □ | □ | □ | □ |
| 14 | Mobilize appropriate personnel and resources to manage a critically ill respiratory patient in the time of COVID-19 | □ | □ | □ | □ | □ |

13. What did you take away from this case and/or how will it change your practice?

____________________________________________________________________

____________________________________________________________________

____________________________________________________________________

14. What specific changes would you make to improve this scenario?

____________________________________________________________________

____________________________________________________________________

15. Other comments or suggestions:

____________________________________________________________________

____________________________________________________________________

____________________________________________________________________

**Thank you for taking the time to complete this survey!**
