# Supplementary material for: COVID-19 Pneumonia and Status Asthmaticus With Respiratory Failure in a Pediatric Patient: A Simulation for Emergency Medicine Providers
Source: MedEdPORTAL. 2022 Jan 21;18:11214. doi: 10.15766/mep_2374-8265.11214 (PMC8776872; doi:10.15766/mep_2374-8265.11214)
Supplement: Supplementary file 1 — Simulation Case.docxEquipment and Medication Checklist.docxLabs and Images.docxDebriefing Guide.docxSurvey.docx [file mep_2374-8265.11214-s001.zip › Educational_Summary_Report_11214.pdf]

MedEdPORTAL® The AAMC Journal of Teaching and Learning Resources

Original Publication Open Access

COVID-19 Pneumonia and Status Asthmaticus With Respiratory Failure in a Pediatric Patient: A Simulation for Emergency Medicine Providers

Hoi See Tsao, MD, MPH*, Mariann Nocera Kelley, MD, Lauren Allister, MD, Robyn Wing, MD, MPH

*Corresponding author: hoisee.tsao@utsouthwestern.edu

Abstract

Introduction: During COVID-19 surges, medical trainees may perform patient care outside typical clinical responsibilities. While respiratory failure in pediatric patients secondary to COVID-19 is rare, it is critical that providers can effectively care for these children while protecting the health care team. Simulation is an important tool for giving learners a safe environment in which to learn and practice these new skills. **Methods:** In this simulation, learners provided care to a 13-year-old male with obesity, COVID-19 pneumonia, status asthmaticus, and respiratory failure. Target learners were pediatric emergency medicine fellows and emergency medicine residents. Providers were expected to identify the signs and symptoms of status asthmaticus, pneumonia, and respiratory failure and demonstrate appropriate evaluation and management while minimizing COVID-19 exposure. Participants completed a postsimulation survey on their satisfaction and confidence in performing the objectives. **Results:** Twenty-eight PGY 1-PGY 6 learners participated in this simulation. The postsimulation survey showed that most learners felt the simulation was effective in teaching the evaluation and management of respiratory failure due to COVID-19 ($M = 5.0$; 95% CI, 4.9-5.0) and was relevant to their work ($M = 5.0$; 95% CI, 5.0-5.0). **Discussion:** Learners felt that the case was effective in teaching the skills needed to care for a child with COVID-19 pneumonia, status asthmaticus, and respiratory failure. Future directions include updating the case with new COVID-19 knowledge and personal protective equipment practices gained over time, using hybrid telesimulation to increase learners' exposure to the case, and adapting the case for other health care providers.

Keywords
Asthma, Pneumonia, Personal Protective Equipment, COVID-19, SARS-CoV-2, Respiratory Failure, Simulation, Pediatric Critical Care Medicine, Pediatric Emergency Medicine, Virtual Learning

Educational Objectives

By the end of this activity, learners will be able to:

1. Establish a differential diagnosis for respiratory distress in a potentially COVID-19-positive pediatric patient.
2. Demonstrate the management of status asthmaticus in a pediatric patient.
3. Identify acute respiratory failure.
4. Anticipate and plan for a difficult airway in a potentially COVID-19-positive patient.
5. Demonstrate the appropriate use of personal protective equipment and resources for aerosolizing procedures with a potentially COVID-19-positive patient.

Citation:
Tsao HS, Kelley MN, Allister L, Wing R. COVID-19 pneumonia and status asthmaticus with respiratory failure in a pediatric patient: a simulation for emergency medicine providers. *MedEdPORTAL*. 2022;18:11214. https://doi.org/10.15766/mep_2374-8265.11214

Introduction

The COVID-19 pandemic has created unprecedented challenges for medical education.[1] While medical providers across specialties have been acquiring new knowledge, altering clinical practice, and learning augmented personal protective equipment (PPE) procedures, simultaneously there has been an urgent need to quickly disseminate this information across clinical and educational settings.[2]

Pediatric emergency medicine (PEM) and emergency medicine (EM) providers and educators face a unique set of circumstances. While pediatric patients have generally had milder symptoms during acute COVID-19 infection compared to adults,[3] acute respiratory failure in pediatric patients secondary to COVID-19

Copyright © 2022 Tsao et al. This is an open-access publication distributed under the terms of the Creative Commons Attribution license.

still occurs, especially in children with comorbid conditions such as asthma.[4] During COVID-19 surges, medical trainees may also need to perform patient care outside their clinical competencies or specialty,[5] such as pediatric providers taking care of adult patients with COVID-19.[6] It is therefore essential that educational curricula tailored to trainees working during the COVID-19 pandemic are created to allow learners to practice taking care of sick COVID-19 patients. Simulation-based training has been shown to be superior to traditional problem-based learning in improving assessment and management skills among medical trainees.[7] Because severely ill pediatric patients with COVID-19 are less commonly seen than adults,[3] simulation provides an opportunity for providers to practice the management of sick pediatric COVID-19 patients in this high-acuity, low-frequency scenario[8] and identify experiential knowledge gaps in a safe setting.

The COVID-19 pandemic has also highlighted the importance of infection control during clinical care. Globally, a large number of health care workers have been infected with COVID-19.[9] Given the persistence of the COVID-19 pandemic, it is important for learners to correctly use PPE and recognize how to modify previously taught methods of managing respiratory illnesses such as asthma or pneumonia to minimize unnecessary infection risk to the health care team, patients, and family members. For example, learners need to learn how to run an effective resuscitation with a limited number of essential personnel to decrease infection exposure, as has been recommended as part of effective resource management during COVID-19.[10] With changing infection-control practices in health care settings and COVID-19 in-person learning restrictions, simulation also provides the advantage of being adaptable to different situations and learning goals[11] and is an effective method for providers to learn new knowledge and practice new skills.

Prior educational curricula developed in the literature in response to COVID-19 include a medical student COVID-19 curriculum,[12] modules on how to use appropriate PPE,[13] bougie-assisted surgical cricothyrotomy for COVID-19 airway management,[14] and many modifications of previously created educational materials for implementation during the COVID-19 pandemic.[14,15] There are also many teaching curricula for status asthmaticus[16,17] or pneumonia[18] with respiratory failure published in *MedEdPORTAL*. However, there are no published curricula addressing the combined management of a pediatric patient with status asthmaticus and COVID-19 pneumonia with respiratory failure with any teaching modality, and none that also incorporate PPE usage.

We present a novel simulation case of a pediatric patient with status asthmaticus and COVID-19 pneumonia progressing to respiratory failure. The primary goal of this case was to teach learners new knowledge and skills, including establishing a differential for respiratory distress in a COVID-19-positive pediatric patient, practicing the management of status asthmaticus, identifying acute respiratory failure, and anticipating and planning for a difficult airway. In addition, trainees learned to protect themselves, their health care team, and other patients from infection by practicing the appropriate use of PPE, managing resources as the team leader to minimize unnecessary COVID-19 exposures, and utilizing effective teamwork and communication while using PPE. This case was targeted towards PEM and EM trainees. It used social learning theory, experiential learning theory, and reflective learning to solidify learning experiences for learners in a safe and effective environment.[19]

Methods

Development

Based on a real clinical case, the simulation case (Appendix A) was developed by PEM physicians to help PEM and EM trainees recognize and manage COVID-19 pneumonia and status asthmaticus in an adolescent patient with obesity. The case was created by PEM faculty members and a PEM fellow with extensive medical education and simulation experience. The critical actions in the simulation case were developed with the purpose of being used by facilitators to identify gaps in participant skills and knowledge to be discussed in the debrief. Prerequisite knowledge included the identification and management of abnormal vital signs, physical exam findings, and respiratory distress. Participants also needed to interpret laboratory results and imaging and to understand institution-specific COVID-19 management protocols and PPE use policies.

Equipment/Environment

The setting was either a pediatric emergency department resuscitation bay or a simulated emergency department resuscitation bay within a medical simulation center. We used SimMan manikins from Laerdal or Gaumard PEDI HAL manikins with padding under a gown to simulate obesity. Vital signs were displayed on a monitor using LLEAP software from Laerdal or Gaumard Vitals software. The equipment and medications available were listed in a checklist (Appendix B). Learners could request laboratory results and diagnostic modalities including chest radiograph and electrocardiogram during the simulation (Appendix C).

Copyright © 2022 Tsao et al. This is an open-access publication distributed under the terms of the Creative Commons Attribution license.

Personnel

Personnel included a simulation technician, a faculty instructor, and a confederate facilitator to play the role of a nurse (Appendix A). Due to COVID-19 in-person learning restrictions, we were restricted to a total of seven people, including learners and facilitators (five learners, one faculty instructor, and one confederate facilitator at one institution, or six learners and one faculty instructor/confederate facilitator at the second institution), in the room with the manikin. The decision to have one or two faculty instructor/confederate facilitators was based on instructor availability. All facilitators were PEM attendings or fellows.

Prior to the session, the faculty instructor and confederate facilitator prepared by reviewing the topics contained within the debriefing guide (Appendix D), including the medical management of status asthmaticus, the most up-to-date resources on the management of COVID-19 respiratory failure, and institution-specific guidelines on PPE use, supplies, and donning/doffing procedures. The instructor and facilitator reviewed their institution's consultant and staffing resources, such as anesthesia or pediatric intensive care unit (PICU), to increase simulation realism. The instructor and facilitator were aware that they had to use the learning objectives and critical actions of the case to facilitate case progression within the available teaching time limit, scaffold learning based on learners' levels of clinical experience through prompting as outlined in the simulation case (Appendix A), and debrief.

Implementation

The simulation activity was implemented during scheduled PEM fellow and EM resident didactics at two different institutions. There were five to six learners in each group. Due to COVID-19 in-person learning restrictions, two learners actively participated in the simulation case while the other participants observed the simulation in the room. Both participating institutions restricted the number of providers at the bedside during aerosolizing procedures to model the case after their institutions' recommended provider number restrictions during COVID-19. The simulated case ran for 20 minutes, with an additional 15-25 minutes for debriefing.

At the start of the simulation, participants were informed by emergency medical services (EMS) that they had brought in a patient with a history of asthma who was in respiratory distress. He was not improving despite the use of his albuterol inhaler at home. EMS noted an oxygen saturation of 88% on room air and placed the patient on 4 L of oxygen via nasal cannula. The two learners were expected to don appropriate PPE prior to entering the room. Upon entry, learners found an obese adolescent in respiratory distress. When participants asked, the confederate facilitator disclosed that the patient lived with an aunt who was COVID-19 positive. Participants were expected to ensure that the nurse had appropriate PPE on and to instruct the nurse to place the patient on the full cardiac monitor and obtain IV access and labs. Participants were expected to recognize the patient's abnormal vital signs (tachycardia, tachypnea, and fever). The patient's examination was notable for obesity, severe respiratory distress, the inability to complete full sentences, tripod positioning, diffuse pulmonary wheeze, and crackles in the right lower lobe.

The patient's vital sign and physical examination abnormalities prompted participants to obtain lab and imaging workup, such as blood gas, electrolytes, complete blood counts, blood culture, chest radiograph, and electrocardiogram (Appendix C). Given the patient's history of asthma, physical examination findings, and labs and imaging findings, participants were expected to manage status asthmaticus and pneumonia with bronchodilators, steroids, intramuscular epinephrine, magnesium, terbutaline, antibiotics, and noninvasive ventilatory support devices such as a bilevel positive airway pressure machine. The nurse asked if therapies such as nebulizers were allowed during COVID-19. Participants were expected to reassure the nurse of the necessity of these medications given the patient's deteriorating clinical status while ensuring appropriate PPE and infection-control measures.

The patient progressed to respiratory failure. Participants were expected to recognize that the patient would likely have a difficult airway due to the patient's obesity, status asthmaticus, and potential COVID-19 infection. In preparing for a difficult intubation, an aerosol-generating procedure, participants were expected to decrease the number of personnel in the room, have an experienced provider with appropriate PPE on intubate with an endotracheal tube filter (depending on institutional resources), and consider calling for help from a PICU or anesthesia provider early.

Learners were expected to recognize that this was a case of status asthmaticus and COVID-19 pneumonia with respiratory failure. The case ended after intubation and sign-out to the PICU. If participants did not recognize respiratory failure and did not intubate the patient within the first 8 minutes, the case progressed to asystolic arrest and ended after one dose of epinephrine and intubation.

Debriefing

Immediately following the simulation, facilitators led the debrief as described in the debriefing guide (Appendix D). Ground rules,

Copyright © 2022 Tsao et al. This is an open-access publication distributed under the terms of the Creative Commons Attribution license.

including that the debrief was a safe and respectful learning environment to encourage participation and optimize learning, were set for participants. Facilitators guided the 15- to 25-minute debrief using the gather, analyze, summarize (GAS) debriefing method.[20] The GAS method was used because it has been adopted by the American Heart Association during debriefings on courses such as Pediatric Advanced Life Support,[21] with which many facilitators are familiar. Alternative debriefing methods such as plus-delta, debriefing with good judgment,[22] or rapid-cycle deliberate practice[23] methods would also be appropriate to use based on facilitator expertise and comfort. Facilitators led the discussion on ways to minimize aerosolizing procedures and COVID-19 exposure risk to health care providers as the team leader. Facilitators also reviewed the differential for respiratory distress in a COVID-19-positive patient, the management of critical COVID-19 disease and status asthmaticus, and how to anticipate and prepare for a difficult airway and call for help from available resources early. Lastly, learners were asked to highlight their take-home points from the simulation session to reinforce learning, and facilitators had a final opportunity to ensure that learning objectives were met.

Assessment

At the end of the session, participants completed the simulation case survey (Appendix E) to evaluate their perceptions of the simulation's effectiveness in achieving its educational objectives. The survey was developed by the authors of this simulation. The survey used 5-point Likert-scale questions regarding whether the educational objectives had been met. The survey also had open-ended questions for participants to explain how the case would change their practice and if they had any recommended changes to improve the simulation case or learning experience.

Results

The simulation was conducted on three occasions at two institutions and facilitated by a total of three PEM attendings across both institutions and one third-year PEM fellow. The simulation had a total of 28 participants across the two institutions. Twenty-three (82%) were EM residents, and five (18%) were PEM fellows, from all levels of training (Table 1).

Based on the postsimulation assessment, residents and fellows felt satisfied with the simulation (Table 2) and reported confidence in completing the simulation objectives (Table 3).

Session feedback from the participants was very positive. One participant stated, "I thought this case was incredibly relevant for the work we do. It was a great opportunity to run through some of the logistics in caring for these patients." This statement was representative of feedback provided by other participants (Table 4). There were very few suggestions for improvement, but they included requests for more in-depth discussion of other treatment modalities for a patient with impending respiratory failure due to status asthmaticus and more realism in simulation to simulate the habitus of a patient with obesity.

Table 1. Learner Level of Training (*N* = 28)

Training Level	No.	%
PGY 1	7	25
PGY 2	10	36
PGY 3	6	21
PGY 4	2	7
PGY 5	2	7
PGY 6	1	4

Discussion

This simulation case taught PEM fellows and EM residents to discuss the differential diagnosis for respiratory distress in a COVID-19-positive pediatric patient, manage critical COVID-19 pneumonia and status asthmaticus, recognize respiratory failure, anticipate a difficult airway, mobilize appropriate resources, and utilize good infection-control practices. While critical respiratory illness has been a less common pediatric presentation of COVID-19 during this pandemic,[3] learning to care for these severely ill pediatric patients in a safe educational environment is paramount as COVID-19 persists. PPE practices have also been a mainstay of COVID-19 care and are therefore an integral skill set to review in a simulation setting. This simulation was a realistic and effective way to practice the management of status asthmaticus, COVID-19 pneumonia, and respiratory failure in a pediatric patient.

This simulation case expands upon prior literature by training providers to apply critical care interventions in the context of COVID-19, a novel disease process, with close attention to infection-control practices for the entire care team. Simulation work has previously been widely used in critical care and

Table 2. Participant Satisfaction With Simulation Case (*N* = 28)

Statement[a]	*Mdn*	*M* (95% CI)	Range
This simulation case is relevant to my work.	5	5.0 (5.0-5.0)	5.0
The simulation case was realistic.	5	4.8 (4.6-4.9)	4.0-5.0
The simulation case was effective in teaching the evaluation and management of respiratory failure in the time of COVID-19.	5	5.0 (4.9-5.0)	4.0-5.0
The debrief created a safe environment.	5	5.0 (4.9-5.0)	4.0-5.0
The debrief promoted reflection and team discussion.	5	5.0 (4.9-5.0)	4.0-5.0

[a]Rated on a 5-point Likert scale (1 = *strongly disagree*, 2 = *disagree*, 3 = *neither agree nor disagree*, 4 = *agree*, 5 = *strongly agree*).

Copyright © 2022 Tsao et al. This is an open-access publication distributed under the terms of the Creative Commons Attribution license.

Table 3. Participant Confidence After Participation in the Simulation Case (*N* = 28)

Statement[a]	*Mdn*	*M* (95% CI)	Range	% Respondents Answering *Confident* or *Very Confident*
After participating in this session, how confident are you in your ability to:				
Manage status asthmaticus.	4	4.3 (4.1-4.5)	4.0-5.0	100
Evaluate the causes of respiratory distress in a potentially COVID-19-positive patient.	4	4.5 (4.3-4.6)	4.0-5.0	100
Evaluate for causes of respiratory distress in a patient with asthma in the time of COVID-19.	4	4.4 (4.2-4.6)	4.0-5.0	100
Identify acute respiratory failure.	5	4.8 (4.6-4.9)	4.0-5.0	100
Anticipate and plan for a difficult airway.	5	4.5 (4.2-4.7)	3.0-5.0	93
Demonstrate appropriate use of personal protective equipment for an aerosolizing procedure in a potentially COVID-19-positive patient.	5	4.7 (4.5-4.9)	4.0-5.0	100
Mobilize appropriate personnel and resources to manage a critically ill respiratory patient in the time of COVID-19.	5	4.7 (4.5-4.9)	4.0-5.0	100

[a]Rated on a 5-point Likert scale (1 = *very not confident,* 2 = *not confident,* 3 = *neutral,* 4 = *confident,* 5 = *very confident*).

trauma training to improve critical care skills,[8] including teaching critical care fellows the management of difficult airway emergencies.[24] Simulation has been shown to improve retention of critical care skills,[25] increase knowledge among critical care providers,[26] and increase learner confidence in critical care and procedural skills.[27] Prior studies have also shown that simulation-based education can translate into greater improvement in patient outcomes compared to no education or nonsimulation instruction.[28] Given the rarity of critical pediatric COVID-19 patients, simulations such as this case aim to teach learners how to recognize and manage a difficult airway while maintaining good infection-control precautions in a sick pediatric patient and can potentially improve patient outcomes among providers who care for children.

Learners in our simulation case also had to broaden their differential for respiratory distress in a patient beyond asthma and pneumonia, including considering acute infection and secondary sequelae from COVID-19. Prior *MedEDPORTAL* curricula have focused on the management of status asthmaticus[16,17] or pneumonia.[18] Prior COVID-19 educational materials have also included an overview of COVID-19 for medical students[12] or have focused on modifying prior educational materials or curricula for delivery in the setting of COVID-19 learning restrictions.[14,15] Our case is novel in presenting a simulation that addresses the combined management of a pediatric patient with status asthmaticus and COVID-19 pneumonia with respiratory failure while adhering to in-person COVID-19 clinical care and learning restrictions.

Overall, this case reinforced critical care skills and knowledge needed to take care of the sick pediatric patient with status asthmaticus, COVID-19 pneumonia, and respiratory failure during a public health crisis.

Recommendations/Lessons Learned

Running this simulation at different institutions highlighted the variations in restrictions and policies related to COVID-19 management. With our increasing understanding of COVID-19 and its effects on pediatric and adolescent patients, varying patient and health care provider vaccination rates, and institutional and community PPE and physical distancing requirement changes, the case will need to be adapted based on updated and institution-specific recommendations and management strategies. This will involve a review of current federal and state recommendations and policies, as well as

Table 4. Sample of Representative Participant Responses

Question	Responses
What did you take away from this case and/or how will it change your practice?	Review of medications for status asthmaticus, particularly epinephrine<br>Keeping a broad differential and avoiding premature closure/anchoring bias<br>Use of appropriate personal protective equipment
What specific changes would you make to improve this scenario?	"None."<br>"It was great."<br>"It was a well-done simulation."<br>"Realistic."<br>Discussion of bilevel positive airway pressure machine to avoid intubation<br>Better simulation of patient's habitus and general appearance
Other comments or suggestions	"I wish we could do more cases like this that are very appropriate with new cases we are seeing."<br>"Very helpful case!"<br>"This case was very well put together and identified an important disease process in a realistic way. It hit all the highlights of an important case. Well organized and well thought out!"

Copyright © 2022 Tsao et al. This is an open-access publication distributed under the terms of the Creative Commons Attribution license.

consultation with institutions' infectious disease and infection prevention/control divisions.

Future iterations of this simulation could include using interdisciplinary teams to increase case realism and learning. For instance, including respiratory therapists would allow for enriched education on best PPE and exposure-mitigation practices during aerosolizing procedures. Hybrid telesimulation could also give more learners exposure to the simulation case and allow for additional facilitators. In addition, while this simulation case has been developed for PEM and EM trainees, it could be adapted for PEM and EM attendings, medical students, and advanced practice practitioners or for pediatrics or family medicine providers.

Limitations

There were several limitations to this simulation case being run in the simulation center at one of the institutions rather than in situ in the emergency department or on the wards. When not in situ, it was difficult to fully practice infection-control specifics, including accessing PPE carts and moving a patient to a negative pressure room. The simulation educational environment and COVID-19 learning restrictions also precluded the team leader from asking for the most experienced nurse or team member when there were a limited number of confederates and other learners actively involved. On the other hand, limiting team members involved in patient care to essential individuals to minimize exposure during aerosolizing procedures created a more realistic simulation experience, as has been seen in code situations during the COVID-19 pandemic.[10]

An additional limitation with this simulation was in its assessment and evaluation methods. Learners' self-reported satisfaction with the simulation case and confidence with the educational objectives were high. Recall bias was minimized by providing participants with the survey immediately after simulation completion to decrease recall period duration. However, these self-reported measures were still subject to social desirability bias. In addition, learners' specific knowledge or skills were not assessed. For example, to better evaluate learners' appropriate use of PPE, checklists on correct PPE usage[29] could have been incorporated into the simulation. Furthermore, while there are data from the literature to suggest that simulation improves patient care outcomes,[28,30] patient outcomes were not directly assessed in this simulation due to the low frequency of pediatric patients with severe COVID-19 illness at the participating institutions during the data-collection phase.

Future Directions

In addition to self-report measures, future iterations of this simulation could include more robust assessment methods and objective evaluations of participants' pre- and postsimulation skills and knowledge, as well as the simulation case's impact on real pediatric patient outcomes in children with status asthmaticus, COVID-19 pneumonia, and/or respiratory failure. These data would help evaluate whether learning from this simulation case translates into closing meaningful clinical performance gaps among medical trainees. More demographic and experiential information in addition to learners' postgraduate year of training could also be collected, such as the number of prior intubations performed. This would better allow readers and educators to determine the generalizability and applicability of this simulation to learners at their respective institutions.

The COVID-19 pandemic continues to generate opportunities to develop more simulation-based education materials, including how to care for the severely ill younger child or infant, the pediatric patient with multiple viral infections (such as rhino/enterovirus and SARS-CoV-2 coinfections), and MIS-C (multisystem inflammatory syndrome in children) patients with or without shock. As simulation has been shown to translate into improved patient outcomes when compared to no education or nonsimulation instruction,[28] further development of pediatric simulation cases in the era of COVID-19 is unquestionably important as we continue to treat patients with this disease. As we learn more about COVID-19, the management of COVID-19 and PPE guidelines may need to be updated over time. This case serves as one example of how to keep our learners' skills and knowledge current based on new information gained.

Appendices

A. Simulation Case.docx
B. Equipment and Medication Checklist.docx
C. Labs and Images.docx
D. Debriefing Guide.docx
E. Survey.docx

All appendices are peer reviewed as integral parts of the Original Publication.

Hoi See Tsao, MD, MPH: Assistant Professor, Division of Pediatric Emergency Medicine, Department of Pediatrics, University of Texas Southwestern Medical Center; ORCID: https://orcid.org/0000-0003-1632-0212

Copyright © 2022 Tsao et al. This is an open-access publication distributed under the terms of the Creative Commons Attribution license.

Mariann Nocera Kelley, MD: Assistant Professor, Division of Pediatric Emergency Medicine, Departments of Pediatrics and Emergency Medicine, University of Connecticut School of Medicine and Connecticut Children's Medical Center; Director of Simulation, University of Connecticut School of Medicine

Lauren Allister, MD: Associate Professor, Division of Pediatric Emergency Medicine, Departments of Emergency Medicine and Pediatrics, Warren Alpert Medical School of Brown University and Rhode Island Hospital/Hasbro Children's Hospital; Associate Program Director of Pediatric Emergency Medicine Fellowship, Warren Alpert Medical School of Brown University

Robyn Wing, MD, MPH: Assistant Professor, Division of Pediatric Emergency Medicine, Departments of Emergency Medicine and Pediatrics, Warren Alpert Medical School of Brown University and Rhode Island Hospital/Hasbro Children's Hospital; Director of Pediatric Simulation, Lifespan Medical Simulation Center

Disclosures
None to report.

Funding/Support
None to report.

Ethical Approval
Reported as not applicable.

References

1. Ferrel MN, Ryan JJ. The impact of COVID-19 on medical education. *Cureus*. 2020;12(3):e7492. https://doi.org/10.7759/cureus.7492
2. Wayne DB, Green M, Neilson EG. Medical education in the time of COVID-19. *Sci Adv*. 2020;6(31):eabc7110. https://doi.org/10.1126/sciadv.abc7110
3. Cruz AT, Zeichner SL. COVID-19 in children: initial characterization of the pediatric disease. *Pediatrics*. 2020;145(6):e20200834. https://doi.org/10.1542/peds.2020-0834
4. Graff K, Smith C, Silveira L, et al. Risk factors for severe COVID-19 in children. *Pediatr Infect Dis J*. 2021;40(4):e137-e145. https://doi.org/10.1097/INF.0000000000003043
5. Goldhamer MEJ, Pusic MV, Co JPT, Weinstein DF. Can Covid catalyze an educational transformation? Competency-based advancement in a crisis. *N Engl J Med*. 2020;383(11):1003-1005. https://doi.org/10.1056/NEJMp2018570
6. Biala D, Siegel EJ, Silver L, Schindel B, Smith KM. Deployed: pediatric residents caring for adults during COVID-19's first wave in New York City. *J Hosp Med*. 2020;15(12):763-764. https://doi.org/10.12788/jhm.3527
7. Steadman RH, Coates WC, Huang YM, et al. Simulation-based training is superior to problem-based learning for the acquisition of critical assessment and management skills. *Crit Care Med*. 2006;34(1):151-157. https://doi.org/10.1097/01.CCM.0000190619.42013.94
8. Hammond J. Simulation in critical care and trauma education and training. *Curr Opin Crit Care*. 2004;10(5):325-329. https://doi.org/10.1097/01.ccx.0000140950.47361.c9
9. Ehrlich H, McKenney M, Elkbuli A. Protecting our healthcare workers during the COVID-19 pandemic. *Am J Emerg Med*. 2020;38(7):1527-1528. https://doi.org/10.1016/j.ajem.2020.04.024
10. Chan PS, Berg RA, Nadkarni VM. Code blue during the COVID-19 pandemic. *Circ Cardiovasc Qual Outcomes*. 2020;13(5):e006779. https://doi.org/10.1161/CIRCOUTCOMES.120.006779
11. Binstadt ES, Walls RM, White BA, et al. A comprehensive medical simulation education curriculum for emergency medicine residents. *Ann Emerg Med*. 2007;49(4):495-504. https://doi.org/10.1016/j.annemergmed.2006.08.023
12. Kochis M, Goessling W. Learning during and from a crisis: the student-led development of a COVID-19 curriculum. *Acad Med*. 2021;96(3):399-401. https://doi.org/10.1097/ACM.0000000000003755
13. Villamagna AH, Bonura EM. Infectious diseases simulation for medical students: experiential instruction on personal protective equipment. *MedEdPORTAL*. 2020;16:11031. https://doi.org/10.15766/mep_2374-8265.11031
14. Asselin M, Lafleur A, Labrecque P, Pellerin H, Tremblay MH, Chiniara G. Simulation of adult surgical cricothyrotomy for anesthesiology and emergency medicine residents: adapted for COVID-19. *MedEdPORTAL*. 2021;17:11134. https://doi.org/10.15766/mep_2374-8265.11134
15. Sukumar S, Zakaria A, Lai CJ, Sakumoto M, Khanna R, Choi N. Designing and implementing a novel virtual rounds curriculum for medical students' internal medicine clerkship during the COVID-19 pandemic. *MedEdPORTAL*. 2021;17:11106. https://doi.org/10.15766/mep_2374-8265.11106
16. Adler M, Trainor J, Siddall V, McGaghie W. Pediatric acute care simulator case: status asthmaticus. *MedEdPORTAL*. 2009;5:1083. https://doi.org/10.15766/mep_2374-8265.1083
17. Reid J, Stone K. Pediatric Emergency Medicine Simulation Curriculum: status asthmaticus. *MedEdPORTAL*. 2014;10:9660. https://doi.org/10.15766/mep_2374-8265.9660
18. Rogers A, Di Rocco J, Soung P. Simulated pediatric respiratory workshop. *MedEdPORTAL*. 2014;10:9693. https://doi.org/10.15766/mep_2374-8265.9693
19. Taylor DCM, Hamdy H. Adult learning theories: implications for learning and teaching in medical education: AMEE Guide no. 83. *Med Teach*. 2013;35(11):e1561-e1572. https://doi.org/10.3109/0142159X.2013.828153

Copyright © 2022 Tsao et al. This is an open-access publication distributed under the terms of the Creative Commons Attribution license.

20. Phrampus PE, O'Donnell JM. Debriefing using a structured and supported approach. In: Levine AI, DeMaria S Jr, Schwartz AD, Sim AJ, eds. *The Comprehensive Textbook of Healthcare Simulation*. Springer; 2013:73-84.

21. Sawyer T, Eppich W, Brett-Fleegler M, Grant V, Cheng A. More than one way to debrief: a critical review of healthcare simulation debriefing methods. *Simul Healthc*. 2016;11(3):209-217. https://doi.org/10.1097/SIH.0000000000000148

22. Gardner R. Introduction to debriefing. *Semin Perinatol*. 2013;37(3):166-174. https://doi.org/10.1053/j.semperi.2013.02.008

23. Perretta JS, Duval-Arnould J, Poling S, et al. Best practices and theoretical foundations for simulation instruction using rapid-cycle deliberate practice. *Simul Healthc*. 2020;15(5):356-362. https://doi.org/10.1097/SIH.0000000000000433

24. Keller JM, Steinbach TC, Adamson R, et al. ICU emergencies simulation curriculum for critical care fellows: the difficult airway. *MedEdPORTAL*. 2018;14:10744. https://doi.org/10.15766/mep_2374-8265.10744

25. Moazed F, Cohen ER, Furiasse N, et al. Retention of critical care skills after simulation-based mastery learning. *J Grad Med Educ*. 2013;5(3):458-463. https://doi.org/10.4300/JGME-D-13-00033.1

26. Boling B, Hardin-Pierce M. The effect of high-fidelity simulation on knowledge and confidence in critical care training: an integrative review. *Nurse Educ Pract*. 2016;16(1):287-293. https://doi.org/10.1016/j.nepr.2015.10.004

27. Cooke JM, Larsen J, Hamstra SJ, Andreatta PB. Simulation enhances resident confidence in critical care and procedural skills. *Fam Med*. 2008;40(3):165-167.

28. Zendejas B, Brydges R, Wang AT, Cook DA. Patient outcomes in simulation-based medical education: a systematic review. *J Gen Intern Med*. 2013;28(8):1078-1089. https://doi.org/10.1007/s11606-012-2264-5

29. Pokrajac N, Schertzer K, Poffenberger CM, et al. Mastery learning ensures correct personal protective equipment use in simulated clinical encounters of COVID-19. *West J Emerg Med*. 2020;21(5):1089-1094. https://doi.org/10.5811/westjem.2020.6.48132

30. Singer BD, Corbridge TC, Schroedl CJ, et al. First-year residents outperform third-year residents after simulation-based education in critical care medicine. *Simul Healthc*. 2013;8(2):67-71. https://doi.org/10.1097/SIH.0b013e31827744f2

Received: May 26, 2021
Accepted: October 23, 2021
Published: January 21, 2022

Copyright © 2022 Tsao et al. This is an open-access publication distributed under the terms of the Creative Commons Attribution license.
